# Applicability of an in-House Saponin-Based Extraction Method in Bruker Biotyper Matrix-Assisted Laser Desorption/Ionization Time-of-Flight Mass Spectrometry System for Identification of Bacterial and Fungal Species in Positively Flagged Blood Cultures

**DOI:** 10.3389/fmicb.2016.01432

**Published:** 2016-09-15

**Authors:** Jung-Yien Chien, Tai-Fen Lee, Shin-Hei Du, Shih-Hua Teng, Chun-Hsing Liao, Wang-Hui Sheng, Lee-Jene Teng, Po-Ren Hsueh

**Affiliations:** ^1^Graduate Institute of Clinical Medicine, College of Medicine, National Taiwan UniversityTaipei, Taiwan; ^2^Departments of Internal Medicine, National Taiwan University Hospital, College of Medicine, National Taiwan UniversityTaipei, Taiwan; ^3^Departments of Laboratory Medicine, National Taiwan University Hospital, College of Medicine, National Taiwan UniversityTaipei, Taiwan; ^4^Departments of Graduate Institute of Clinical Laboratory Sciences and Medical Biotechnology, National Taiwan UniversityTaipei, Taiwan; ^5^Department of Graduate Institute of Biomedical Sciences, Chang Gung UniversityTao-Yuan, Taiwan; ^6^Bruker Taiwan Co., Ltd.Taipei, Taiwan; ^7^Department of Internal Medicine, Far Eastern Memorial HospitalTaipei, Taiwan

**Keywords:** MALDI-TOF MS, Bruker Biotyper system, Vitek MS system, flagged blood cultures, performance

## Abstract

We used an in-house saponin-based extraction method to evaluate the performance of the Bruker Biotyper matrix-assisted laser desorption/ionization time-of-flight mass spectrometry (MALDI-TOF/MS) system for the identification of bacteria and fungi in 405 positively flagged blood culture bottles. Results obtained from MALDI-TOF/MS were compared with those obtained using conventional phenotypic identification methods. Of the 405 positively flagged blood culture bottles, 365 showed monomicrobal growth and were correctly identified to the species (72.1%) or genus (89.6%) level using the Bruker Biotyper system. The remaining 40 positively flagged blood culture bottles showed polymicrobial growth. Of them, 82.5% (*n* = 33) of the isolates were correctly identified to the species level and 92.5% (*n* = 37) to the genus level using the Bruker Biotyper system. The overall accuracy of identification to the genus level in flagged blood cultures was 89.5% for Gram-positive organisms, 93.5% for Gram-negative pathogens and 71.9% for fungi. Confidence scores were ≥1.500 for 307 (75.8%) bottles, ≥1.700 for 249 (61.5%) bottles and ≥2.000 for 142 (35.1%) bottles. None of the yeast cultures yielded scores ≥1.700. Using an identification-score cutoff of ≥1.500, the MALDI Biotyper correctly identified 99.2% of Gram-positive bacteria, 97.6% of Gram-negative bacteria and 100% of yeast isolates to the genus level and 77.6% of Gram-positive bacteria, 87.1% of Gram-negative bacteria and 100.0% of yeast isolates to the species level. The overall rate of identification using our protocol was 89.9% (364/405) for genus level identification and 73.1% (296/405) for species level identification. Yeast isolates yielded the lowest confidence scores, which compromised the accuracy of identification. Further optimization of the protein extraction procedure in positive blood cultures is needed to improve the rate of identification.

## Introduction

Bloodstream infections are a leading cause of admission to intensive care units and carry a high mortality rate. Identification of the causative microorganism(s) is central to the treatment of bloodstream infections, and clinical outcome can be greatly improved by the timely administration of appropriate antimicrobial agents (Deen et al., 2012; Lai et al., 2014; Bassetti et al., 2015).

Matrix-assisted laser desorption/ionization time-of-flight mass spectrometry (MALDI-TOF/MS) techniques are now routinely used for the direct identification of microorganisms from agar cultures and positively flagged blood cultures (Moussaoui et al., 2010; Lagace-Wiens et al., 2012; Martiny et al., 2012). These techniques provide definitive identification of pathogens causing bloodstream infections 18–48 h earlier than conventional methods. Identification of pathogens using the MALDI-TOF/MS system has markedly reduced the rate of administering improper antimicrobial agents, has contributed to decreased morbidity and mortality rates and is associated with reduced hospital costs for patients with bloodstream infections (Loonen et al., 2012; Martiny et al., 2013). A number of purification methods are available to prepare samples for MALDI-TOF/MS analysis such as differential centrifugation, lysis centrifugation, pre-incubation on sold media and the SepsiTyper™ kit (Schubert et al., 2011; Saffert et al., 2012; March-Rossello et al., 2013). Of those methods, processing of specimens using the SepsiTyper™ kit has been shown to result in highly accurate identification rates; however, the kit is not widely used because of its high cost (Buchan et al., 2012; Lagace-Wiens et al., 2012).

In this study, we used an in-house saponin-based extraction method that was modified from a previous study (Martiny et al., 2012) to evaluate the performance of the Bruker Biotyper (Bruker Daltonics) MALDI TOF-MS system for the identification of bacteria and fungi in positively flagged blood culture bottles.

## Materials and methods

### Blood cultures

All positive cultures preserved in Bactec Plus Aerobic/F bottles or Bactec Anaerobic Lytic/10 bottles (Becton-Dickinson Microbiology Systems, Sparks, MD, USA) that had been obtained from patients treated for bloodstream infections at the National Taiwan University Hospital (NTUH) during the period October 13, 2014 to January 15, 2015 were evaluated by conventional phenotypic methods and the MALDI TOF-MS Biotyper system. For each patient, only the first positive blood culture broth was included in the study. Time to positivity (TTP) of each positively flagged blood cultures was evaluated. Microbial identification using the MALDI Biotyper was performed directly from positive blood culture broth and from subsequent colonies on Tryticase soy agar with 5% sheep blood agar (BAP) or chocolate agar after overnight culture.

### Processing of flagged positive blood cultures by the MALDI biotyper

The in-house saponin-based extraction method used in this study followed the protocol described by Martiny et al. with slight modifications (Martiny et al., 2012). Briefly, 1 mL of positive blood sample was added to 200 μL of a 5% saponin lysis solution. The tube was thoroughly vortexed for 10 s and then centrifuged for 1 min at 13,000 g. The supernatant was discarded, the pellet was repeatedly washed by pipetting with 1 mL of de-ionized water and then the solution was centrifuged for 1 min at 13,000 g. The supernatant was discarded and the pellet was subjected to a formic acid extraction method for MALDI Biotyper analysis as described below. All microbial species reported by the MALDI Biotyper were recorded even when identification score values were <2.000 (genus-level identification or no reliable identification).

### Performance of the MALDI biotyper from subcultured colonies

For analysis by the MALDI Biotyper system on BAP, two to three colonies were transferred to a 1.5-ml screw-cap Eppendorf tube containing 300 μl of distilled water and then mixed with 900 μl of ethanol by pipetting. The suspension was pelleted by centrifugation at 13,000 rpm for 2 min, evaporated to dryness, and then reconstituted in 50 μl of 70% formic acid. After incubation for 30 s, 50 μl of acetonitrile (Sigma-Aldrich) was added. The suspension was then centrifuged at 13,000 rpm for 2 min. Then, 1.0 μl of the supernatant was applied to a 96-spot polished steel target plate (Bruker Daltonik GmbH, Bremen, Germany) and dried. A saturated solution of 1.0 μl of MALDI matrix (HCCA; Bruker Daltonik GmbH, Bremen, Germany) was applied to each sample and dried. Measurements were performed with the Bruker Microflex™ LT MALDI-TOF MS system (Bruker Daltonik GmbH, Bremen, Germany) using FlexControlTM software with Compass Flex Series version 1.3 software and a 60 Hz nitrogen laser (337 nm wavelength). Spectra were collected in the linear positive mode in a mass range covering 1960–20,132 m/z. Spectra ranging from the mass-to-charge ratio (m/z) 2000–20,000 were analyzed using Bruker Biotyper automation control and the Bruker Biotyper 3.1 software and library (DB 5627 with 5627 entries). Identification scores of ≥2.000 indicated species-level identification, scores ranging from 1.700 to 1.999 indicated genus-level identification, and scores of <1.700 indicated no reliable identification. All isolates with discrepant identification results between phenotypic and Bruker Biotyper methods were retested twice.

### Species identification by conventional phenotypic methods

Two commercial biochemical identification systems, namely the Vitek 2 system (bioMe′rieux, Marcy l'Etoile, France) and the Phoenix system (Becton-Dickinson Microbiology Systems), were routinely used for species identification of positive blood cultures. The Phoenix system NMIC/ID-72 and PMIC/ID-30 were used for identification of *Streptococcus* species and *Aeromonas* species, respectively. For identification of other bacterial organisms, the Vitek 2 system (Vitek 2 GN and Vitek 2 GP cards) was used. For identification of yeasts, the Vitek 2 yeast identification card (Vitek 2 Yeast ID) was used.

### Comparison of identification results

**Figure 1:**
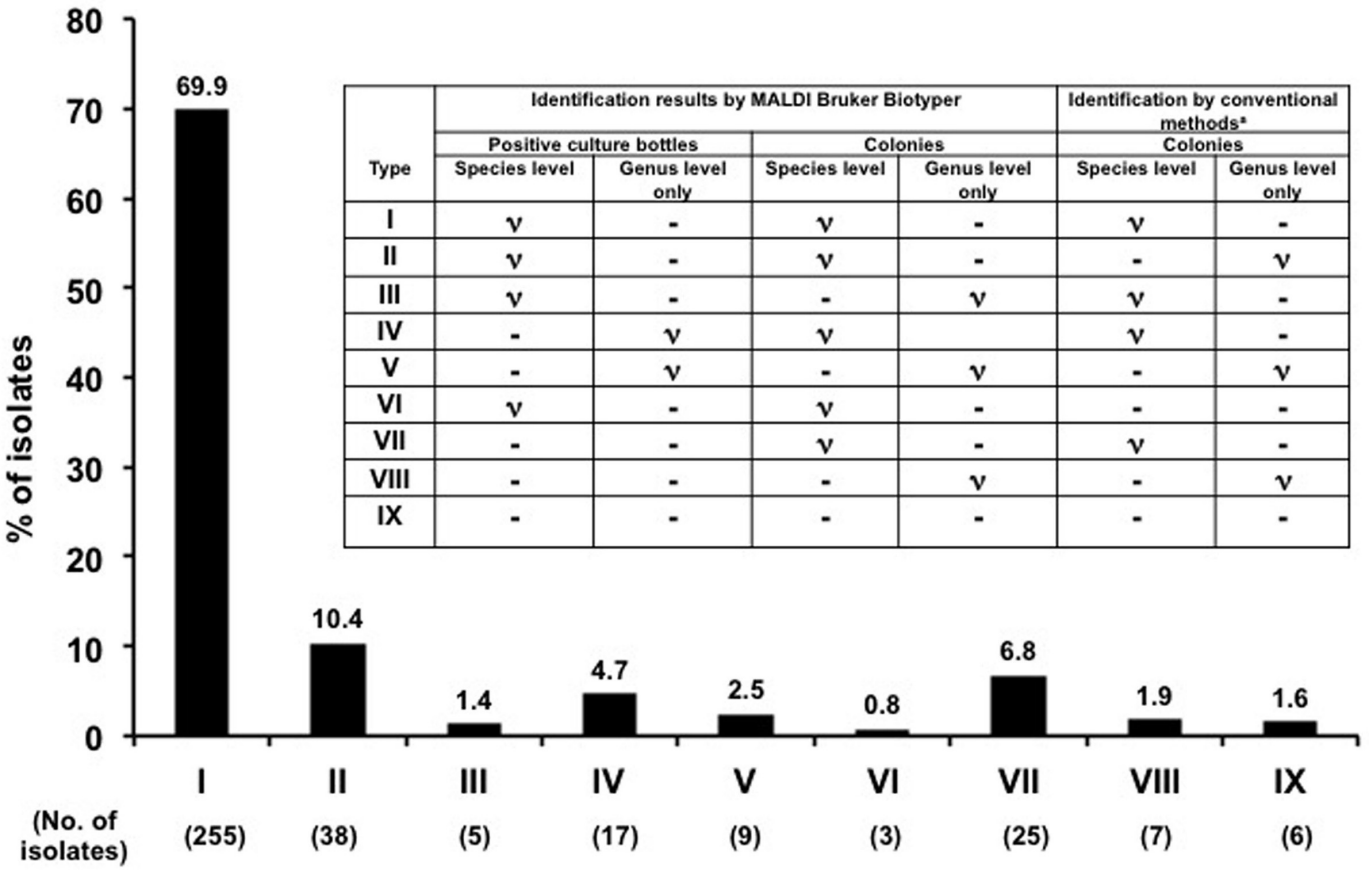
**Identification of flagged blood culture bottles by MALDI Biotyper**. Comparisons of microbial identification results in 365 monomicrobial flagged blood culture bottles by three methods: flagged culture bottles and subcultured growth colonies from flagged culture bottles by the MALDI Biotyper and conventional identification systems. Patterns of identification (I–IX) were defined based on the identity of identification to the species or genus level among the three methods.

The clinical microbiology laboratory at the NTUH routinely reported the identification results based on those obtained by conventional phenotypic identification methods. In this study, the identification results obtained by the MALDI Biotyper system from positive blood culture bottles were compared with those obtained by the MALDI Biotyper and conventional phenotypic identification methods from sub-cultured colonies. We used the identification results by the MALDI Biotyper from subcultured growth colonies to resolve the discrepancy between results by the MALDI Biotyper system directly from positive blood culture bottles and conventional phenotypic identification methods. The rates of concordant results for identification of organisms to the genus and species levels were calculated. Types of identification (I–IX) were defined based on the results of identification to the species or genus level among three methods (Figure 1).

### Turn-around time (TAT) of blood cultures

TAT of blood cultures was defined as the time interval between collection of blood from patients and identification results from positive blood cultures reported by the central laboratory. The maximum time allowed to collect blood samples, transport the blood culture bottles to the laboratory, and upload the culture bottles into the Bactec FX system was 2 h. Following the alarming of positive blood cultures, around 2 h was need to stain the positive culture broths, prepare and perform the MALDI-TOF MS analysis (around 30 min), and report the identification results via laboratory and hospital information systems. The TAT to identify specific organisms directly from flagged blood cultures by the MALDI Biotyper was calculated as the average TTP for specific organisms in positive blood cultures plus 4 h. The TAT to identify specific organisms isolated from blood cultures identified by the MALDI Biotyper and conventional phenotypic identification methods from sub-cultured colonies was 16–24 h longer than the TAT to identify specific organisms in flagged blood cultures using the MALDI Biotyper system.

## Results

During the study period, a total of 405 positive blood cultures, including 365 monomicrobal and 40 polymicrobial positive blood cultures that had been determined by the conventional identification systems were evaluated by MALDI-TOF MS.

### Discrepancy analysis

Table 1 shows the TTP data and rates of concordant identification to the genus and species levels for 365 monomicrobial blood cultures in flagged culture bottles and subcultured colonies by the MALDI Biotyper and by conventional phenotypic identification methods. All isolates of *Staphylococcus aureus* (*n* = 23), *Enterococcus faecalis* (*n* = 6), and *Pseudomonas aeruginosa* (*n* = 8) were correctly identified by the phenotypic methods and MALDI-TOF. Greater than 90% concordance was obtained for *Escherichia coli* (*n* = 69) and *Klebsiella pneumoniae* (*n* = 31). Among the 29 isolates of *Candida* species, 21 (72.4%) were identified to the genus level and 13 (44.8%) were identified to the species level in flagged culture bottles by the MALDI Biotyper and by conventional phenotypic identification methods in subcultured colonies. Only one of seven isolates of *C. tropicalis* in flagged culture bottles was identified correctly by the MALDI Biotyper.

**Table 1:** **Comparison of identification results in 365 monomicrobial positive blood cultures by conventional identification systems with those by the MALDI Biotyper in flagged culture bottles and subcultured growth colonies**.

**Identification by conventional methods, Phoenix or Vitek 2^a^**		**Flagged blood cultures**	**Concordant identification by the MALDI Bruker Biotyper directly from positive blood culture bottles or from subcultured growth colonies on BAP or chocolate agar sub-cultured from positive blood culture bottles, no. of isolates (%)**							
**Species**	**No. of isolates**	**TTP, h mean (range)**	**Bottles**				**Colonies**			
			**Species level**	**Score value mean (range)**	**Genus level**	**Score value mean (range)**	**Species level**	**Score value (range)**	**Genus level**	**Score value (range)**
*S. aureus*	23	18.8 (6.6–85.4)	23 (100.0)	1.83 (1.162–2.135)	23 (100.0)	1.83 (1.162–2.135)	23 (100.0)	2.24 (1.852–2.474)	23 (100.0)	2.24 (1.852–2.474)
CoNS	59	27.8 (8.9–103.1)	45 (76.3)	1.75 (1.273–2.312)	57 (96.6)	1.75 (1.259–2.312)	49 (83.1)	2.06 (1.782–2.283)	59 (100.0)	2.06 (1.586–2.338)
*S. epidermidis*	25	24.6 (13.6–42.9)	22 (88.0)	1.65 (1.273–1.985)	24 (96.0)	1.64 (1.273–1.985)	25 (100.0)	2.05 (1.809–2.199)	25 (100.0)	2.05 (1.809–2.199)
*S. capitis*	12	29.7 (13.7–60.3)	11 (91.7)	1.87 (1.384–2.312)	12 (100.0)	1.82 (1.259–2.312)	12 (100.0)	2.10 (1.992–2.264)	12 (100.0)	2.10 (1.992–2.264)
*S. hominis*	4	42.1 (18.1–103.1)	3 (75.0)	1.80 (1.546–2.163)	3 (75.0)	1.80 (1.546–2.163)	3 (75.0)	2.14 (2.083–2.201)	4 (100.0)	2.11 (2.03–2.201)
Other CoNS	18	27.6 (8.9–95.5)	9 (50.0)	1.83 (1.382–2.043)	18 (100.0)	1.8 (1.361–2.081)	9 (50.0)	2.01 (1.782–2.283)	18 (100.0)	2.02 (1.586–2.338)
*Streptococcus* species	19	12.96 (5.9–20.7)	11 (57.9)	1.78 (1.269–2.216)	17 (89.5)	1.79 (1.269–2.216)	14 (73.7)	2.18 (1.966–2.372)	19 (100.0)	2.17 (1.966–2.372)
*S. agalactiae*	5	11.04 (6.7–19)	5 (100.0)	1.91 (1.613–2.216)	5 (100.0)	1.91 (1.613–2.216)	5 (100.0)	2.16 (2.035–2.269)	5 (100.0)	2.16 (2.035–2.269)
*S. anginosus*	2	19.25 (17.9–20.6)	1 (50.0)	1.44 (1.438)	1 (50.0)	1.44 (1.438)	2 (100.0)	2.19 (2.187–2.194)	2 (100.0)	2.19 (2.187–2.194)
*S. mitis*	2	16.10 (15.4–16.8)	1 (50.0)	1.82 (1.815)	2 (100.0)	1.81 (1.811–1.815)	1 (50.0)	2.28 (2.282)	2 (100.0)	2.15 (2.022–2.282)
*S. oralis*	2	12.50 (11.7–13.3)	0 (0.0)		2 (100.0)	1.93 (1.841–2.015)	1 (50.0)	1.97 (1.966)	2 (100.0)	2.09 (1.966–2.205)
*S. pyogenes*	2	11.45 (10.3–12.6)	2 (100.0)	1.95 (1.83–2.062)	2 (100.0)	1.95 (1.83–2.062)	2 (100.0)	2.29 (2.205–2.372)	2 (100.0)	2.29 (2.205–2.372)
*S. pneumoniae*	1	14.50 (14.5–14.5)	0 (0.0)		0 (0.0)		1 (100.0)	2.08 (2.079–2.079)	1 (100.0)	2.08 (2.079–2.079)
Others	5	11.60 (5.9–20.7)	2 (40.0)	1.44 (1.269–1.607)	5 (100.0)	1.61 (1.269–2.038)	2 (40.0)	2.17 (2.143–2.199)	5 (100.0)	2.16 (2.105–2.235)
*Enterococcus* species	26	17.3 (3.1–62.9)	22 (84.6)	1.93 (1.173–2.431)	24 (92.3)	1.95 (1.173–2.431)	24 (92.3)	2.31 (1.959–2.529)	26 (100.0)	2.29 (1.959–2.529)
*E. faecium*	17	18.5 (8.6–62.9)	15 (88.2)	2.00 (1.304–2.431)	15 (88.2)	2.00 (1.304–2.431)	17 (100.0)	2.30 (1.959–2.529)	17 (100.0)	2.30 (1.959–2.529)
*E. faecalis*	6	19 (9.7–34.4)	6 (100.0)	1.78 (1.173–2.129)	6 (100.0)	1.78 (1.173–2.129)	6 (100.0)	2.33 (2.264–2.439)	6 (100.0)	2.33 (2.264–2.439)
*E. gallinarum*	2	4.2 (3.1–5.2)	0 (0.0)		2 (100.0)	2.10 (2.066–2.134)	0 (0.0)		2 (100.0)	2.11 (2.104–2.117)
*E. casseliflavus*	1	12.8 (12.8)	1 (100.0)	1.89 (1.890)	1 (100.0)	1.89 (1.890)	1 (100.0)	2.30 (2.303)	1 (100.0)	2.30 (2.303)
*Bacillus* species	9	12.1 (8.8–18.5)	2 (22.2)	2.00 (1.874–2.13)	9 (100.0)	1.83 (0.948–2.169)	3 (33.3)	1.89 (1.662–2.13)	9 (100.0)	1.91 (1.332–2.233)
*E. coli*	69	13.7 (1.3–93.5)	67 (97.1)	2.07 (1.306–2.351)	67 (97.1)	2.07 (1.306–2.351)	68 (98.6)	2.25 (1.94–2.474)	69 (100.0)	2.24 (1.94–2.474)
*K. pneumoniae*	31	16.1 (6.8–61.9)	30 (96.8)	1.92 (1.364–2.319)	30 (96.8)	1.92 (1.364–2.319)	31 (100.0)	2.23 (1.517–2.447)	31 (100.0)	2.23 (1.517–2.447)
*Enterobacter* species	12	14.2 (6.5–35.9)	7 (58.3)	1.92 (1.547–2.183)	11 (91.7)	1.88 (1.547–2.183)	9 (75.0)	2.15 (1.945–2.402)	12 (100.0)	2.14 (1.945–2.402)
*E. cloacae*	11	14.6 (6.5–35.9)	6 (54.5)	1.87 (1.547–2.156)	10 (90.9)	1.85 (1.547–2.156)	8 (72.7)	2.11 (1.945–2.232)	11 (100.0)	2.12 (1.945–2.232)
*E. aerogenes*	1	9.8 (9.8)	1 (100.0)	2.183 (2.183)	1 (100.0)	2.183 (2.183)	1 (100.0)	2.402 (2.402)	1 (100.0)	2.402 (2.402)
*Citrobacter* species	3	10.6 (9.1–13.3)	3 (100.0)	2.031 (1.949–2.133)	3 (100.0)	2.031 (1.949–2.133)	3 (100.0)	2.134 (2.083–2.178)	3 (100.0)	2.134 (2.083–2.178)
*C. koseri*	2	11.4 (9.4–13.3)	2 (100.0)	1.98 (1.949–2.011)	2 (100.0)	1.98 (1.949–2.011)	2 (100.0)	2.16 (2.141–2.178)	2 (100.0)	2.16 (2.141–2.178)
*C.‘braakii*	1	9.1 (9.1)	1 (100.0)	2.13 (2.133)	1 (100.0)	2.13 (2.133)	1 (100.0)	2.08 (2.083)	1 (100.0)	2.08 (2.083)
*Salmonella* species	5	13.5 (9.2–17)	5 (100.0)	1.95 (1.694–2.156)	5 (100.0)	1.95 (1.694–2.156)	5 (100.0)	2.19 (1.912–2.303)	5 (100.0)	2.19 (1.912–2.303)
*Aeromonas* species	2	10.4 (9.9–10.8)	0 (0.0)		2 (100.0)	1.74 (1.736–1.752)	0 (0.0)		2 (100.0)	1.20 (2.171–2.23)
*A. hydrophila*	1	9.9 (9.9)	0 (0.0)		1 (100.0)	1.75 (1.752)	0 (0.0)		1 (100.0)	2.23 (2.230)
*A. sobria*	1	10.8 (10.8)	0 (0.0)		1 (100.0)	1.74 (1.736)	0 (0.0)		1 (100.0)	2.17 (2.171)
*P. aeruginosa*	8	14.5 (8.7–18.6)	8 (100.0)	2.09 (1.49–2.35)	8 (100.0)	2.09 (1.49–2.35)	8 (100.0)	2.35 (2.096–2.495)	8 (100.0)	2.35 (2.096–2.495)
*Acinetobacter* species	13	21.4 (7.2–98.4)	5 (38.5)	1.75 (1.364–2.142)	11 (84.6)	1.57 (1.226–2.142)	5 (38.5)	2.17 (1.879–2.354)	13 (100.0)	2.03 (1.631–2.404)
*A. baumannii*	11	20.6 (7.2–98.4)	4 (36.4)	1.76 (1.364–2.142)	9 (81.8)	1.59 (1.226–2.142)	4 (36.4)	2.20 (1.879–2.354)	11 (100.0)	2.04 (1.631–2.404)
*A. ursingii*	1	11.1 (11.1)	1 (100.0)	1.72 (1.718)	1 (100.0)	1.72 (1.718)	1 (100.0)	2.03 (2.033)	1 (100.0)	2.03 (2.033)
*A. junii*	1	40.9 (40.9)	0 (0.0)		1 (100.0)	1.25 (1.251)	0 (0.0)		1 (100.0)	1.94 (1.943)
*S. maltophilia*	4	33.2 (16.7–65.6)	2 (50.0)	1.43 (1.328–1.527)	2 (50.0)	1.43 (1.328–1.527)	3 (75.0)	1.95 (1.926–1.993)	4 (100.0)	1.98 (1.926–2.047)
*Burkholderia* species	5	30.0 (12.6–57.3)	2 (40.0)	2.14 (2.031–2.258)	5 (100.0)	1.88 (1.487–2.258)	3 (60.0)	1.94 (1.54–2.258)	5 (100.0)	2.32 (2.23–2.274)
*B. cenocepacia*	1	12.6 (12.6)	1 (100.0)	2.03 (2.031)	1 (100.0)	2.03 (2.031)	1 (100.0)	2.34 (2.341)	1 (100.0)	2.34 (2.341)
*B. cepacia* complex	4	34.4 (18–57.3)	1 (25.0)	2.26 (2.258)	4 (100.0)	1.85 (1.487–2.258)	2 (50.0)	1.90 (1.54–2.258)	4 (100.0)	2.31 (2.23–2.374)
*Chryseobacterium* species	6	24.7 (15.1–45.3)	1 (16.7)	1.37 (1.368)	4 (66.7)	1.72 (1.368–1.922)	0 (0.0)		4 (66.7)	2.09 (1.751–2.232)
*C. indologenes*	4	18.7 (15.1–20.1)	1 (25.0)	1.37 (1.368)	4 (100.0)	1.72 (1.368–1.922)	0 (0.0)		4 (100.0)	2.09 (1.751–2.232)
Others	2	36.7 (28.1–45.3)	0 (0.0)		0 (0.0)		0 (0.0)		0 (0.0)	
*Elizabethkingia* species	12	15.6 (7.6–32.2)	7 (58.3)	1.76 (1.556–1.961)	10 (83.3)	1.75 (1.359–1.981)	10 (83.3)	1.64 (1.359–1.961)	12 (100.0)	2.03 (1.843–2.283)
*E. meningoseptica*	11	16.11 (7.6–32.2)	7 (63.6)	1.76 (1.556–1.961)	9 (81.8)	1.74 (1.359–1.981)	10 (90.9)	1.64 (1.359–1.961)	11 (100.0)	2.03 (1.843–2.283)
*E. miricola*	1	9.9 (9.9)	0 (0.0)		1 (100.0)	1.80 (1.799)	0 (0.0)		1 (100.0)	2.00 (1.998)
*Bacteroides* species	4	41.1 (27.4–47.9)	3 (75.0)	1.74 (1.529–2.068)	3 (75.0)	1.74 (1.529–2.068)	2 (50.0)	1.96 (1.943–1.974)	3 (75.0)	1.91 (1.825–1.974)
*B. thetaiotaomicron*	2	36.2 (27.4–45)	2 (100.0)	1.84 (1.62–2.068)	2 (100.0)	1.84 (1.62–2.068)	2 (100.0)	1.96 (1.943–1.974)	2 (100.0)	1.96 (1.943–1.974)
*B. fragilis*	1	44.1 (44.1)	0 (0.0)		0 (0.0)		0 (0.0)		0 (0.0)	
*B. caccae*	1	47.9 (47.9)	1 (100.0)	1.53 (1.529)	1 (100.0)	1.53 (1.529)	0 (0.0)		1 (100.0)	1.83 (1.825–1.825)
*Candida* species	29	79.7 (6.1–1114.1)	13 (44.8)	1.40 (1.134–1.673)	21 (72.4)	1.30 (0.829–1.673)	27 (93.1)	1.24 (0–1.673)	29 (100.0)	1.85 (1.422–2.296)
*C. ablicans*	10	52.2 (6.1–129.1)	5 (50.0)	1.44 (1.315–1.673)	8 (80.0)	1.32 (1.054–1.673)	9 (90.0)	1.35 (1.054–1.673)	11 (00.0)	1.90 (1.748–2.099)
*C. glabrata*	7	52.1 (28.2–82.1)	4 (57.1)	1.34 (1.248–1.491)	6 (85.7)	1.25 (1.048–1.491)	7 (100.0)	1.87 (1.528–2.088)	7 (100.0)	1.87 (1.528–2.088)
*C. tropicalis*	7	32.1 (14.5–63.3)	1 (14.3)	1.57 (1.569)	2 (28.6)	1.45 (1.333–1.569)	7 (100.0)	1.77 (1.422–2.068)	7 (100.0)	1.77 (1.422–2.068)
*C. parapsilopsis*	4	295.4 (15.7–1114.1)	2 (50.0)	1.34 (1.134–1.537)	4 (100.0)	1.25 (0.829–1.537)	3 (75.0)	1.80 (1.544–1.974)	4 (100.0)	1.73 (1.536–1.974)
*C. krusei*	1	19.1 (19.1)	1 (100.0)	1.37 (1.37)	1 (100.0)	1.37 (1.37)	1 (100.0)	2.30 (2.296)	1 (100.0)	2.30 (2.296)

a *For identification of Streptococcus species and Aeromonas species, the Phoenix system was used and for identification of other organisms, the Vitek 2 system was used*.

*CoNS, coagulase-negative staphylococci; BAP, blood agar plates, TTP, Time-to-positive blood cultures*.

One isolate from a flagged positive culture bottle was incorrectly identified as *S. aureus* by the MALDI Biotyper (identification score, 1.006); however, that isolate was correctly identified as *Micrococcus* species from subcultured colonies by the MALDI Biotyper (identification score, 2.181) and the Vitek 2 system. One isolate from a flagged positive culture bottle was incorrectly identified as *K. pneumoniae* by the MALDI Biotyper (identification score, 1.185); however, that isolate was subsequently correctly identified as *E. coli* from subcultured colonies by the MALDI Biotyper (identification score, 2.247) and the Vitek 2 system. Two isolates of *S. maltophilia* in positive blood culture bottles were correctly identified by the MALDI Biotyper.

### Monomicrobial growth

Table 2 summarizes 37 incorrect identification results (types of identification VII, VIII, and IX) in monomicrobial growth colonies by the MALDI Biotyper in flagged blood culture bottles in comparison with those by MALDI Biotyper in subculture growth colonies from flagged culture bottles and conventional identification systems. The identification results obtained by the MALDI Biotyper and the conventional identification system in subcultured growth colonies were 83.8% compatible at the species level (*n* = 23) and the genus level (*n* = 8).

**Table 2:** **Summary of 37 incorrect identification results (types of identification VII, VIII, and IX) in monomicrobial flagged culture bottles by the MALDI Bruker Biotyper in comparison with those by the MALDI Biotyper in subcultured growth colonies from flagged culture bottles and conventional identification systems (see Figure 1 for description of identification types)**.

**No**.	**TTP (h)**	**Gram staining findings in positive blood cultures**	**Identification results by MALDI Biotyper**				**Identification results by conventional methods**	**Patterns of identification**
			**Bottle, organism (top three matches)**	**Score value**	**Colony, Organism (best match)**	**Score value**		
1.	11.5	GNB	*Sphingobium chlorophenolicum*	1.223	*Acinetobacter baumannii*	2.30	*A. baumannii*	VII
			*Aromatoleum terpenicum*	1.133				
			*Moraxella bovis*	1.129				
2.	22.1	Yeasts	*LactoB. sharpeae*	1.427	*Candida albicans*	1.79	*C. albicans*	VII
			*L. sharpeae*	1.267				
			*L. crispatus*	1.247				
3.	77.1	Yeasts	*A. terpenicum*	1.293	*C. albicans*	1.748	*C. albicans*	VII
			*A. bremensis*	1.188				
			*Staphylococcus aureus*	1.128				
4.	62.1	Yeasts	*A. anaerobicus*	1.159	*C. glabrata*	1.628	*C. glabrata*	VII
			*A. anaerobicus*	1.140				
			*S. chlorophenolicum*	1.116				
5.	15.7	Yeasts	*Riemerella columbina*	1.196	*C. tropicalis*	1.422	*C. tropicalis*	VII
			*L. antri*	1.111				
			*C. tropicalis*	1.064				
6.	22.1	Yeasts	*Penicillium digitatum*	1.135	*C. tropicalis*	1.756	*C. tropicalis*	VII
			*C. albicans*	1.070				
			*C. albicans*	0.99				
7.	63.3	Yeasts	*Pseudomonas putida*	1.235	*C. tropicalis*	1.591	*C. tropicalis*	VII
			*A. ramosus*	1.126				
			*P. putida*	1.124				
8.	58.2	Yeasts	No peaks found	–	*C. tropicalis*	1.671	*C. tropicalis*	VII
9.	14.5	Yeasts	*A. terpenicum*	1.347	*C. tropicalis*	2.068	*C. tropicalis*	VII
			*A. terpenicum*	1.302				
			*Arthrobacter bergerei*	1.270				
10.	61.6	GPB	*Mannheimia haemolytica*	1.172	*Corynebacterium riegelii*	2.099	*C. riegelii*	VII
			*P. savastanoi*	1.158				
			*Bacillus wakoensis*	1.119				
11.	18.3	GNB	*Weissella halotolerans*	1.367	*Elizabethkingia meningoseptica*	2.014	*E. meningoseptica*	VII
			*Flavobacterium johnsoniae*	1.266				
			*L. gastricus*	1.248				
12.	19.6	GNB	*P. savastanoi*	1.395	*E. miricola*	1.849	*E. meningoseptica*	VII
			*Stenotrophomonas maltophilia*	1.162				
			*S. pasteuri*	1.157				
13.	15.1	GNB	*M. haemolytica*	1.233	*Enterobacter cloacae*	1.945	*E. cloacae*	VII
			*Rhodococcus rhodochrous*	1.097				
			*P. putida*	1.094				
14.	16.7	GPB	*Agromyces italicus*	1.228	*Enterococcus faecium*	2.15	*E. faecium*	VII
			*L. agilis*	1.181				
			*E. faecium*	1.145				
15.	19.1	GPC	*Streptococcus acidominimus*	1.122	*E. faecium*	2.417	*E. faecium*	VII
			*L. parabuchneri*	1.106				
			*Brachybacterium faecium*	1.093				
16.	11.9	GNB	*Klebsiella pneumoniae*	1.185	*Escherichia coli*	2.247	*E. coli*	VII
			*P. putida*	1.178				
			*S. pasteuri*	1.135				
17.	13.1	GNB	*Clostridium perfringens*	1.979	*E. coli*	2.187	*E. coli*	VII
			*C. perfringens*	1.916				
			*C. perfringens*	1.819				
18.	10.6	GNB	*E. hormaechei*	1.853	*K. pneumoniae*	2.107	*K. pneumoniae*	VII
			*E. asburiae*	1.816				
			*E. kobei*	1.805				
19.	56.7	GPC	*M. haemolytica*	1.300	*Parvimonas micra*	2.215	*P. micra*	VII
			*Aeromonas eucrenophila*	1.158				
			*A. veronii*	1.119				
20.	77.9	GPC	*L. antri*	1.054	*P. micra*	1.774	*P. micra*	VII
			*S. lentus*	1.051				
			*A. italicus*	1.022				
21.	16.8	GPC	*L. paracasei* ssp. *tolerans*	1.307	*S. epidermidis*	2.038	*S. epidermidis*	VII
			*L. paracasei* ssp. *tolerans*	1.213				
			*Janthinobacterium lividum*	1.209				
22.	14.5	GPC	*S. aureus*	1.363	*S. pneumoniae*	2.079	*S. pneumoniae*	VII
			*L. gasseri*	1.137				
			*C. lusitaniae*	1.102				
23.	32.1	Yeasts	*C. albicans*	1.087	*Trichosporon asahii*	1.862	*T. asahii*	VII
			*C. albicans*	1.068				
			*Malassezia pachydermatis*	1.012				
24.	65.6	GNB	*E. miricola*	1.014	*S. maltophilia*	1.993	*S. maltophilia*	VII
			*E. meningoseptica*	1.01				
			*E. meningoseptica*	0.992				
25.	18.5	GPB	*Mycobacterium szulgai*	1.123	*C. striatum*	2.343	*C*. species	VIII
			*C. dubliniensis*	1.106				
			*A. bremensis*	1.102				
26.	92.5	GPC	*S. aureus*	1.006	*M. luteus*	2.181	*M*. species	VIII
			*S. aureus*	1.003				
			*S. pasteuri*	0.995				
27.	82.7	GPC	*A. terpenicum*	1.444	*Micrococcus luteus*	2.217	*M*. species	VIII
			*A. terpenicum*	1.242				
			*Azoarcus species*	1.232				
28.	46.7	Not visible	*P. savastanoi*	1.265	*M. luteus*	2.005	*M*. species	VIII
			*P. putida*	1.15				
			*P. congelana*	1.064				
29.	15	GNB	*S. chlorophenolicum*	1.185	*A. baylyi*	1.742	*A. baumannii*	VIII
			*C. chauvoei*	1.153				
			*A. radioresistens*	1.102				
30.	103.1	GPC	*A. bremensis*	1.449	*S. pettenkoferi*	2.03	*S. hominis*	VIII
			*A. terpenicum*	1.436				
			*L. sharpeae*	1.29				
31.	31.2	GNB	*M. canis*	0.971	*S. rhizophila*	2.047	*S. maltophilia*	VIII
			*M. canis*	0.947				
			*S. intermedius*	0.938				
32.	44.1	GPB	*L. crispatus*	1.266	*Thauera aromatica*	1.285	*Bacteroides fragilis*	IX
			*Tissierella praeacuta*	1.225				
			*P. brassicacearum*	1.208				
33.	45.3	GNB	*P. putida*	1.185	*S. rhizophila*	2.062	*Chryseobacterium* species	IX
			*L. sharpeae*	1.114				
			*L. alimentaurius*	1.102				
34.	28.1	GPB	*T. aromatica*	1.369	*A. cumminsii*	1.849	*Chryseobacterium* species	IX
			*A. cumminsii*	1.276				
			*S. faeni*	1.271				
35.	33.2	GPB	*S. herbicidovorans*	1.167	*A. polychromogenes*	1.813	*C*. species	IX
			*Sphingomonas adhaesiva*	1.126				
			*S. adhaesiva*	1.126				
36.	78.5	GPC	*L. mucosae*	1.31	*Propionibacterium avidum*	1.422	*Eggerthella lenta*	IX
			*A. globiformis*	1.305				
			*P. antarctica*	1.267				
37.	79.8	GPB	*Actinomyces graevenitzii*	1.099	*A. nosocomialis*	1.594	*E. lenta*	IX
			*R. erythropolis*	1.062				
			*Vibrio mimicus*	1.042				

*TTP, time-to-positive blood culture; GPC, Gram-positive cocci; GPB, Gram-positive bacilli; GNB, Gram-negative bacilli*.

In general, among the monomicrobal positive blood cultures, the MALDI Biotyper identified 89.6% (identification types I–V) of organisms directly from positive blood cultures to the species (72.1%) or genus levels compared with by conventional phenotypic methods from sub-cultured colonies of positive blood cultures (Figure 1).

### Polymicrobial growth

Among the 40 flagged blood culture bottles with polymicrobial growth, two organisms were isolated from 38 (95%) of the bottles and three organisms were isolated from two (5%) of the bottles (Table 3). Among these 40 flagged blood culture bottles, the MALDI Biotyper correctly identified one of the isolated organisms to the species level in 33 (82.5%) bottles and to the genus level in 37 (92.5%). The results of one bottle (no. 25) identified by the MALDI Biotyper as containing *Aeromonas jandaei* (best-matched organism identified) and *E. coli* (second-matched organism identified) were compatible with the identification results obtained by conventional phenotypic methods. The results of three bottles (no. 7, 22, and 28) identified by the MALDI Biotyper as comprising *Acinetobacter nosocomialis, Aromatoleum terpenicum*, and *K. pneumonia*, respectively, differed from those obtained by the phenotypic methods. The results of species identification by the MALDI Biotyper (best-match organisms) from 34 bottles (85.0%) and from colonies on BAP or chocolate agar sub-cultured from positive blood culture bottles were identical to those obtained by conventional phenotypic methods.

**Table 3:** **Comparisons of microbial identification results in 40 polymicrobial flagged blood culture bottles: flagged culture bottles and subculture growths from flagged culture bottles by the MALDI Biotyper and conventional identification systems**.

**No**.	**Gram staining findings in positive blood cultures**	**Time-to positive blood cultures (hours)**	**Species identification by MALDI-TOF from flagged positive blood culture bottles**				**Species identification by MALDI Bruker Biotyper directly from growths (colonies) on BAP or chocolate agar sub-cultured from positive blood culture bottles (best match)**		**Species identification by conventional methods, Phoenix or Vitek 2^a^**
			**Best match**		**Second match**		**Species**	**Score value**	
			**Species**	**Score value**	**Species**	**Score value**			
1.	GPC	191.1	*S. epidermidis*	1.619	*S. epidermidis*	1.57	*S. epidermidis*	1.939	*S. haemolyticus*
							*S. parasanguinis*	2.014	*S. parasanguinis*
2.	GNB	9.5	*E. gergoviae*	1.759	*E. gergoviae*	1.73	*E. gergoviae*	2.085	*E. gergoviae*
							*A. guillouiae*	1.756	*Acinetobacter baumannii* complex
3.	GPC	11.3	*Enterococcus faecalis*	1.651	*E. faecalis*	1.635	*E. faecalis*	2.314	*E. faecalis*
							*Candida tropicalis*	1.972	*C. tropicalis*
4.	GNB	4.6	*Escherichi. coli*	2.106	*E. coli*	1.997	*E. coli*	2.364	*E. coli*
							*E. coli*	2.471	*E. coli*
5.	GPC	10.1	*S. epidermidis*	1.58	*S. epidermidis*	1.578	*S. cohnii* subspecies *urealyticus*	2.486	*S. cohnii* subspecies *urealyticus*
							*S. epidermidis*	2.186	*S. epidermidis*
6.	GNB	8.3	*Enterobacter cancerogenus*	1.835	*E. asburiae*	1.8	*E. asburiae*	2.244	*E. cloacae*
							*A. baumannii*	2.143	*A. baumannii* complex
7.	GNB	6.6	*A. nosocomialis*	1.544	*Brevibacterium linens*	1.18	*E. kobei*	2.027	*E. cloacae*
							*Klebsiella oxytoca*	2.303	*K. oxytoca*
8.	GPC	14.9	*E. faecium*	2.068	*E. faecium*	2.066	*E. faecium*	2.199	*E. faecium*
							*Stenotrophomonas maltophilia*	2.225	*S. maltophilia*
9.	GNB	11.8	*Elizabethkingia meningoseptica*	1.917	*E. miricola*	1.765	*E. miricola*	2.167	*E. miricola*
							*A. baumannii*	2.137	*A. baumannii* complex
10.	GPC	20.3	*Staphylococcus hominis*	1.848	*S. hominis*	1.834	*S. hominis*	2.037	*S. hominis*
							*S. capitis*	2.165	*S. capitis*
11.	GNB	8.1	*E. coli*	2.14	*E. coli*	2.123	*E. coli*	2.449	*E. coli*
							*K. pneumonia*	2.04	*K. pneumonia*
12.	GNB	10.3	*E. coli*	2.117	*E. coli*	2.054	*E. coli*	2.164	*E. coli*
							*K. pneumonia*	2.27	*K. pneumonia*
13.	GPC	11.1	*E. faecalis*	1.707	NRI	1.653	*E. faecalis*	2.214	*E. faecalis*
							*Proteus mirabilis*	2.317	*P. mirabilis*
14.	GNB	3.8	*K. pneumoniae*	1.686	*E. cloacae*	1.669	*K. pneumonia*	2.382	*K. pneumoniae*
							*E. cloacae*		*E. cloacae*
15.	GPC	21.3	*Streptococcus agalactiae*	1.502	*S. agalactiae*	1.369	*S. agalactiae*	2.147	*S. agalactiae*
							*P. mirabilis*	2.253	*P. mirabilis*
16.	GNB	6.4	*E. coli*	2.193	*E. coli*	2.118	*E. coli*	1.74	*E. coli*
							*K. pneumonia*	2.321	*K. pneumonia*
17.	GNB	11.4	*E. coli*	2.108	*E. coli*	2.095	*E. coli*	2.32	*E. coli*
							*Pseudomonas aeruginosa*	1.851	*P. aeruginosa*
18.	GNB	8	*A. baumannii*	1.442	*A. baumannii*	1.108	*A. nosocomialis*	2.342	*A. baumannii* complex
							*S. haemolyticus*	2.144	*S. haemolyticus*
							*S. epidermidis*	2.007	*S. epidermidis*
19.	GNB	15.1	*E. coli*	1.701	NRI	1.695	*E. coli*	1.975	*E. coli*
							*P. mirabilis*	2.172	*P. mirabilis*
20.	GPC, GNB	6.6	*K. pneumoniae*	1.703	*E. coli*	1.689	*E. coli*	2.274	*E. coli*
							*K. pneumoniae*	2.354	*K. pneumonia*
							*E. faecalis*	2.355	*E. faecalis*
21.	GNB	7.8	*E. coli*	2.038	*E. coli*	2.029	*E. coli*	2.1	*E. coli*
							*K. pneumonia*	2.09	*K. pneumonia*
22.	GPB, yeasts	25.1	*Aromatoleum terpenicum*	1.432	*Lactobacillus fermentum*	1.185	*Candida albicans*	1.878	*C. albicans*
							*Burkholderia vietnamiensis*	2.179	*Burkholderia cepacia* complex
23.	GNB	7.1	*K. pneumoniae*	2.047	*K. pneumoniae*	1.928	*K. pneumonia*	2.226	*K. pneumonia*
							*E. coli*	1.93	*E. coli*
							*Shewanella algae*	1.82	*S. algae*
24.	GNB	9.6	*Aeromonas jandaei*	1.734	*A. veronii*	1.729	*A. caviae*	1.928	*A. hydrophila*
							*E. coli*	2.359	*E. coli*
25.	GNB	11.3	*A. jandaei*	1.817	*E. coli*	1.77	*A. caviae*	1.978	*A. hydrophila*
							*E. coli*	2.316	*E. coli*
26.	GNB	11.7	*A. baumannii*	1.948	*A. baumannii*	1.697	*A. baumannii*	2.068	*A. baumannii*
							*S. epidermidis*	1.915	*S. epidermidis*
27.	GNB	11.1	*A. baumannii*	2.041	*A. baumannii*	1.608	*A. baumannii*	2.002	*A. baumannii*
							*Acinetobacter* species	1.935	*A. lwoffii*
28.	GNB	12.2	*K. pneumoniae*	1.728	*E. aerogenes*	1.686	*E. aerogenes*	2.334	*E. aerogenes*
							*E. aerogenes*	2.234	*E. aerogenes*
29.	GPC	19.1	*S. aureus*	1.616	*S. aureus*	1.479	*S. aureus*	2.279	*S. aureus*
							*E. faecium*	2.236	*E. faecium*
30.	GNB	9.6	*Plesiomonas shigelloides*	1.927	*P. shigelloides*	1.915	*P. shigelloides*	2.223	*P. shigelloides*
							*E. coli*	2.243	*E. coli*
31.	GNB, GPC	11.6	*E. coli*	2.326	*E. coli*	2.28	*E. coli*	2.065	*E. coli*
							*S. epidermidis*		*S. epidermidis*
32.	Yeasts	15.5	*E. faecium*	1.633	*E. faecium*	1.516	*E. faecium*	1.87	*E. faecium*
							*C. parapsilosis*	2.304	*C. parapsilosis*
33.	GNB	16.8	*K. pneumoniae*	2.097	*K. pneumoniae*	2.018	*K. pneumonia*	2.213	*K. pneumoniae*
							*P. aeruginosa*	2.295	*P. aeruginosa*
34.	GNB	22.3	*E. coli*	2.225	*E. coli*	2.221	*E. coli*	2.426	*E. coli*
							*Neisseria mucosa*	1.901	*N. sicca*
35.	GNB	9.9	*E. coli*	2.159	*E. coli*	2.152	*E. coli*	2.206	*E. coli*
							*K. pneumonia*	2.246	*K. pneumonia*
36.	GNB,GPC	10.3	*E. coli*	2.098	*E. coli*	2.066	*E. coli*	2.275	*E. coli*
							*S. anginosus*	1.996	*S. anginosus*
37.	GPC	17.8	*S. capitis*	2.075	*S. capitis*	1.593	*S. capiti*	1.778	*S. capitis*
							*Clostridium perfringens*	2.109	*C. perfringens*
38.	GNB	16.1	*K. pneumoniae*	1.658	*K. pneumoniae*	1.597	*A. nosocomialis*	2.380	*A. baumannii*
							*A. nosocomialis*	2.311	*A. baumannii*
39.	GPC	14.1	*Serratia ureilytica*	2.203	*S. marcescens*	2.201	*E. faecalis*	2.318	*E. faecalis*
							*S. marcescens*	2.136	*S. marcescens*
40.	GPC	17.1	*E. faecium*	1.574	*E. faecium*	1.554	*E. faecium*	2.38	*E. faecium*

a *For identification of Streptococcus species and Aeromonas species, the Phoenix system was used and for identification of other organisms, the Vitek 2 system was used*.

*GPC, Gram-positive cocci; GNB, Gram-negative bacilli; No, no bacteria or yeasts were visible; NRI, no reliable identification*.

### Concordant identification and TAT

Table 4 shows that among 162 bottles with Gram-positive organisms and 200 bottles with Gram-negative organisms, the MALDI Biotyper correctly identified 145 (89.5%) and 187 (93.5%), respectively, to the genus level and 111 (68.5%) and 161 (80.5%), respectively, to the species level. Among 32 bottles with organisms that stained positive for yeasts, the results of identification to the species level for 23 (71.9%) obtained by the MALDI Biotyper were compatible with those obtained by conventional phenotypic methods. Among the bottles with score values ≥1.500, the MALDI Biotyper correctly identified 99.2% (124/125) of the bottles with Gram-positive organisms, 97.6% (166/170) of the bottles with Gram-negative organisms and 100% (5/5) of the bottles with yeasts to the genus level and 77.6% (97/125) of the bottles with Gram-positive organisms, 87.1% (148/170) of the bottles with Gram-negative organisms and 100.0% (5/5) of the bottles with yeasts to the species level.

**Table 4:** **Concordant identification to the species or genus level by the MALDI Biotyper directly from 405 flagged blood culture bottles and conventional identification systems according to Gram staining results and identification scores**.

**Identification scores value**	**Gram stain findings in positive blood cultures**											
	**Gram-positive**			**Gram-negative**			**Yeast**			**Other^a^**		
	**No**.	**Species level no. (%)**	**Genus level no. (%)**	**No**.	**Species level no. (%)**	**Genus level no. (%)**	**No**.	**Species level no. (%)**	**Genus level no. (%)**	**No**.	**Species level no. (%)**	**Genus level no. (%)**
All	162	111 (68.5)	145 (89.5)	200	161 (80.5)	187 (93.5)	32	16 (50.0)	23 (71.9)	11	8 (72.7)	9 (81.8)
<1.399	30	11 (36.7)	16 (53.3)	21	6 (28.6)	12 (57.1)	24	14 (1.7)	16 (66.7)	3	1 (33.3)	2 (66.7)
1.400–1.499	7	3 (42.9)	5 (71.4)	9	7 (77.8)	9 (100.0)	3	1 (33.3)	2 (66.7)	1	0 (0.0)	0 (0.0)
1.500–1.599	15	12 (80.0)	14 (93.3)	10	7 (70.0)	9 (90.0)	3	3 (100.0)	3 (100.0)	2	2 (100.0)	2 (100.0)
1.600–1.699	19	14 (73.7)	19 (100.0)	7	6 (85.7)	6 (85.7)	2	2 (100.0)	2 (100.0)	0	–	–
1.700–1.999	48	37 (77.1)	48 (100.0)	57	41 (71.9)	55 (96.5)	0	–	–	2	2 (100.0)	2 (100.0)
≧2.000	43	34 (79.1)	43 (100.0)	96	94 (97.9)	96 (100.0)	0	–	–	3	3 (100.0)	3 (100.0)
≧1.400	132	100 (75.8)	129 (97.7)	179	155 (86.6)	175 (97.8)	8	6 (75.0)	7 (87.5)	8	7 (87.5)	7 (87.5)
≧1.500	125	97 (77.6)	124 (99.2)	170	148 (87.1)	166 (97.6)	5	5 (100.0)	5 (100.0)	7	7 (100.0)	7 (100.0)
≧1.600	110	85 (77.3)	110 (100.0)	160	141 (88.1)	157 (98.1)	2	2 (100.0)	2 (100.0)	5	5 (100.0)	5 (100.0)
≧1.700	91	71 (78.0)	91 (100.0)	153	135 (88.2)	151 (98.7)	0	–	–	5	5 (100.0)	5 (100.0)

a *No bacteria or yeasts were visible or no mixed organisms were found*.

**Figure 2:**
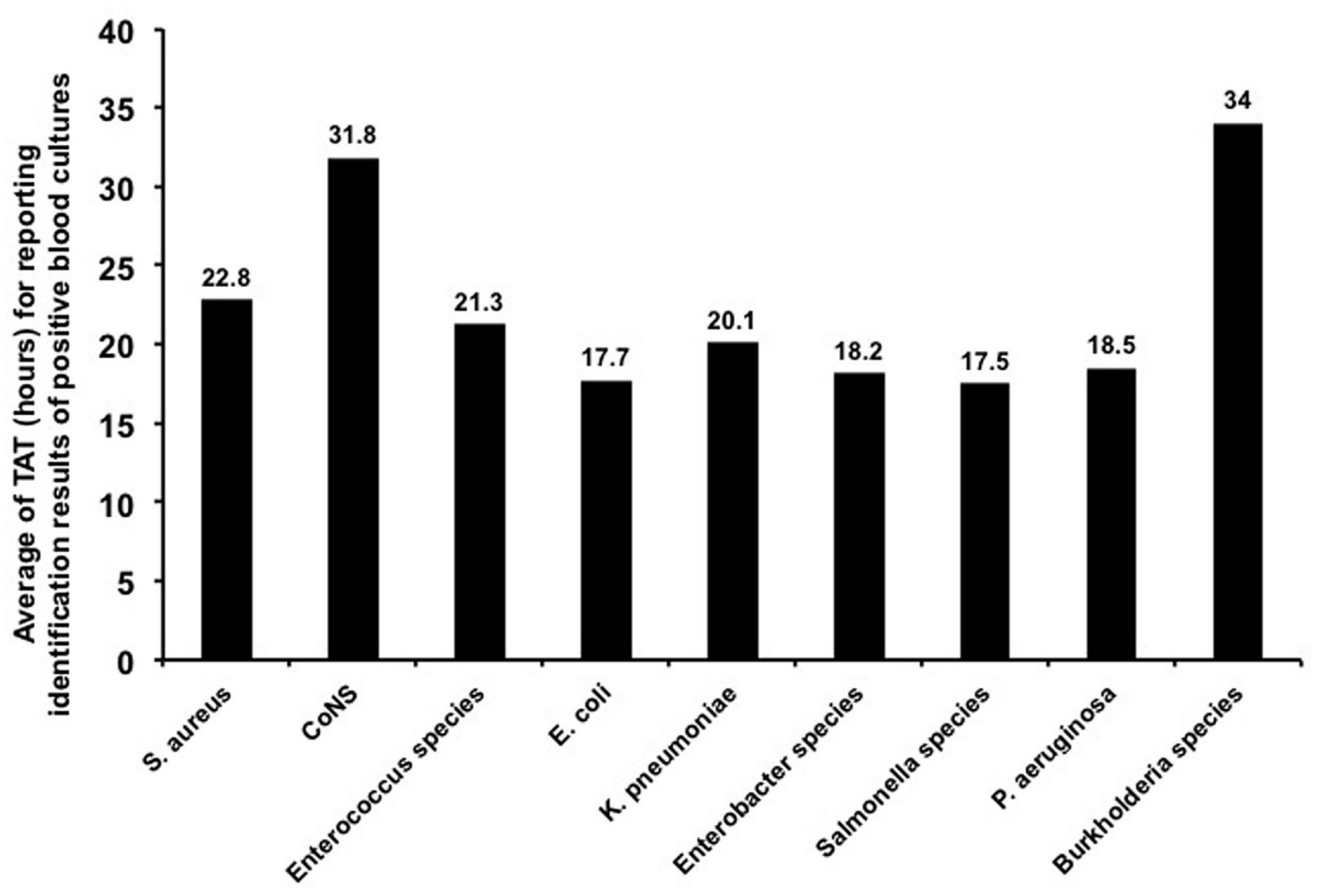
**Turn-around time (TAT) of positive blood cultures**. TAT of blood cultures for several main microorganisms (no. of isolates ≥5) from positive blood cultures with concordant identification results of ≥90% with conventional phenotypic methods. TAT of blood cultures was defined as the time interval between blood cultures collected from patients and laboratory reporting of identification results from positive blood cultures.

Figure 2 shows the TAT of blood cultures for several of the main microorganisms (species with no. of isolates ≥5) in positive blood cultures with ≥90% concordant identification results between the MALDI Biotyper in flagged blood cultures and conventional phenotypic methods. The mean TAT for species-level identification in flagged blood cultures by the MALDI Biotyper was 22.8 h (range, 10.6–89.4 h) for *S. aureus*, 17.7 h (range, 5.3–97.5 h) for *E. coli*, 20.1 h (range, 10.8–65.9 h) for *K. pneumoniae*, and 18.5 h (range, 12.7–22.6 h) for *P. aeruginosa*.

## Discussion

We used an in-house saponin-based extraction method to evaluate the performance of the Bruker Biotyper MALDI-TOF/MS system for the identification of bacteria and fungi in 405 positively flagged blood culture bottles. Results obtained from MALDI-TOF/MS were compared with those obtained using conventional phenotypic identification methods. Among 365 bottles with monomicrobal growth, the microorganisms were correctly identified to the species (72.1%) or genus (89.6%) level. Moreover, among 40 polymicrobial cultures, the MALDI Biotyper correctly identified at least one organism to the species level in 33 (82.5%) positive blood culture bottles and to the genus level in 37 (92.5%) bottles.

MALDI-TOF MS is a useful modality for identifying bacterial species directly from positive blood cultures. For accurate results, however, protein extraction methods are necessary to reduce the number of background peaks caused by non-bacterial proteins in blood culture bottles. Schubert et al. (2011) and Juiz et al. (2012) reported that the Sepsityper kit was superior to centrifugation methods for protein extraction in BACTEC bottles. More recently, some studies have demonstrated that saponin-based extraction leads to results identical to if not better than those obtained by the Sepsityper kit. Using BacT/ALERT anaerobic positive blood cultures, Meex et al. (2012) reported no significant difference in the rates of correct species-level identification between the Sepsityper kit (67%) and a saponin-based extraction method (66%). Working from BACTEC bottles, Martiny et al. also found that the rates of correct species-level identification from positive blood culture bottles were similar between the Sepsityper kit (68.4%) and an in-house saponin-based extraction method (73.7%) (Martiny et al., 2012).

Rapid identification of organisms to the genus level is essential, especially for organisms with predictable resistance (such as *Enterobacter* sp., *Acinetobacter* sp., *Pseudomonas* sp., and *Stenotrophomonas* sp.) (Chen et al., 2013; Davey et al., 2013; Huang et al., 2013; Nagel et al., 2014). We found that the MALDI Biotyper provided good genus-level identification results. The sensitivity of the system for correctly identifying pathogens to the genus level was 89.6% in monomicrobal cultures, and the overall accuracy of identification to the genus level in positive blood cultures was 89.5% for Gram-positive organisms, 93.5% for Gram-negative organisms and 71.9% for yeasts. These findings are in agreement with those reported in previous studies, which showed that the accuracy of MALDI-TOF was higher for Gram-negative organisms than for Gram-positive organisms (Lagace-Wiens et al., 2012; Meex et al., 2012).

The rate of correct identification by MALDI-TOF MS is largely dependent on confidence score. Among the 405 positive blood culture bottles investigated in this study, 307 (75.8%) had confidence scores ≧1.500, 249 (61.5%) had scores ≧1.700 and 142 (35.1%) had confidence scores ≧2.000; none of the yeast cultures had scores ≧1.700. With a cutoff score of ≧1.700 (Bruker's recommended criteria for genus identification), the MALDI Biotyper correctly identified 78.0% of Gram-positive organisms and 88.2% of Gram-negative organisms to the species level and 100 and 98.7%, respectively, to the genus level. In a recent study, Lagace-Wiens et al. found that the cutoff values could be lowered without compromising accuracy when MALDI-TOF is applied directly to positive blood cultures (Lagace-Wiens et al., 2012). Working from BACTEC bottles, Schubert et al. (2011) also demonstrated the possibility of accepting species-level identifications in blood culture bottles with low scores (≧1.500) if the first three proposed results were identical. In BacT/ALERT anaerobic blood culture bottles, Meex et al. further showed that Bruker's recommended criteria could be expanded to avoid the exclusion of a significant percentage of correct identifications, mainly among Gram-positive bacteria (Meex et al., 2012). Similarly, with a lower cutoff score of 1.500, we found that the MALDI Biotyper could correctly identify Gram-positive organisms, Gram-negative organisms and yeasts to the species (77.6, 87.1, and 100%, respectively) or genus level (99.2, 97.6, and 100%, respectively) in blood culture bottles. Thus, for identification to the genus level, we propose that the cutoff value can be lowered to ≧1.500 without compromising the reliability of the identification results (accuracy>95%).

Although some studies have reported that MALDI-TOF can accurately identify yeasts to the species level (Spanu et al., 2012; Won et al., 2013), we found that most blood cultures containing yeast isolates had low confidence scores and lower rates of correct identification than blood cultures containing bacteria only. Buchan et al reported similar findings (Buchan et al., 2012). This may be because the extraction protocol used in our study and in that by Buchan et al was not optimized for recovery of yeasts. However, with a modified cut off value of 1.500, yeasts in all 5 bottles with confidence scores ≧1.500 were correctly identified to the species level. An additional extraction step would be required to obtain more reliable identification results for yeasts with confidence scores <1.500 (Martiny et al., 2012).

In polymicrobial cultures, MALDI-TOF MS typically yields a single identification confidence score for the pre-dominant species (Lagace-Wiens et al., 2012) and is unable to identify multiple organisms. Although one study reported that it may be possible to identify multiple organisms with different Gram staining reactions in polymicrobial cultures (Ferroni et al., 2010), we found that the MALDI Biotyper correctly identified only two organisms in 2 (5.0%) bottles among 40 polymicrobial cultures. In contrast, organisms in a polymicrobial culture could be identified to the species level in 34 bottles (85.0%) from subcultures on BAP. This emphasizes the importance of subculturing positive cultures for definitive identification of organisms in polymicrobial cultures.

Rapid identification of bloodstream pathogens is important so that appropriate antibiotic treatment can be administered (Vlek et al., 2012). Using the MALDI Biotyper to identify specimens prepared by the Sepsityper kit, Buchan et al. found that the median TAT (from blood collection to species identification) was 23–83 h faster than routine methods for Gram-positive isolates and 34–51 h faster for Gram-negative isolates (Buchan et al., 2012). Egli et al. also found that the median TAT was 27.4 (25.8–29.3) h using MALDI-TOF (Egli et al., 2015). With our in-house saponin-based extraction protocol, we found that the mean TAT for identification of Gram-positive organisms (*S. aureus*, coagulase-negative staphylococci and *Enterococcus* species) was 21–32 h and that the mean TAT for identification of Gram-negative isolates was less than 20 h. The TAT of conventional phenotypic identification methods from sub-cultured colonies were 37–56 h for Gram-positive isolates and 36–52 h for Gram-negative isolates which were 16–24 h longer than that of MALDI Biotyper system.

## Summary

In this study, we evaluated the performance of the MALDI Biotyper with an in-house saponin-based method for routine identification of isolates directly from positive blood culture bottles. Our protocol yielded a genus-level identification rate of 89.9% (364/405) and a species-level identification rate of 73.1% (296/405). Confidence scores for yeasts were significantly lower than those for Gram-positive and Gram-negative isolates and compromised the accuracy of identification. Further optimization of the protein extraction procedure in positive blood cultures is needed to improve the rate of identification.

## Author contributions

JC, TL, and SD conceived and designed the experiments, performed the experiments, analyzed the data, and wrote the paper. JC, TL, SD, ST, CL, and WS performed the experiments and analyzed the data. JC, TL, SD, ST, CL, WS, and PH read and approved the final version of the manuscript.

### Conflict of interest statement

The authors declare that the research was conducted in the absence of any commercial or financial relationships that could be construed as a potential conflict of interest.
